# Identification of differentially expressed genes, lncRNAs and miRNAs which are associated with tumor malignant phenotypes in hepatoblastoma patients

**DOI:** 10.18632/oncotarget.22181

**Published:** 2017-10-31

**Authors:** Sida Liu, Fujing Xie, Xiaohong Xiang, Sinan Liu, Shunbin Dong, Kai Qu, Ting Lin

**Affiliations:** ^1^ Department of The Second General Surgery, Shaanxi Provincial People’s Hospital, Xi’an 710068, China; ^2^ Department of Pediatrics, Liaocheng People’s Hospital, Taishan Medical College, Liaocheng 252000, China; ^3^ Department of Hepatobiliary Surgery, The First Affiliated Hospital of Xi'an Jiaotong University, Xi'an 710061, China; ^4^ Department of Surgical Intensive Care Units, The First Affiliated Hospital of Xi'an Jiaotong University, Xi'an 710061, China

**Keywords:** hepatoblastoma, lncRNA, miRNA, mRNA, malignant phenotype

## Abstract

Hepatoblastoma (HB) is one of the most common hepatic malignancies in the pediatric population. HB are composed of a variety of tumors, which derived from different origins and had varying clinical outcomes. However, the unclear underlying mechanisms of HB limited exploring novel biomarkers and effective therapeutic targets. We searched microarray datasets on Gene Expression Omnibus (GEO) database and selected GSE75271 and GSE75283 datasets for comprehensive analysis. Weighted gene correlation network analysis (WGCNA) was employed to identify genes which were associated with tumor malignant phenotypes, including HB subtypes, Cairo classification and tumor stage. Coexpression analysis of identified genes was also performed and lncRNA-miRNA-mRNA network was finally conducted. Our results showed that a total of 22 lncRNAs, 13 miRNAs and 66 mRNAs were identified to be associated with tumor malignant phenotypes. Mechanistically, these molecules might promote the malignant phenotypes via regulating metabolic pathways. Among of them, 6 miRNAs (hsa-miR-106b, hsa-miR-130b, hsa-miR-19a, hsa-miR-19b, hsa-miR-20a and hsa-miR-301a), 8 lncRNAs (NR_102317, XR_245338, XR_428373, XR_924945, XR_929728, XR_931611, XR_935074 and XR_946696), and 6 mRNAs (*EGFR, GAREM, INSIG1, KRT81, SAR1B* and *SDC1*) were selected to conduct a lncRNA-miRNA-mRNA network. Taken together, our findings provide evidence for exploring molecular mechanisms of HB. Those identified malignant phenotype-associated molecules might be potential biomarkers and anti-cancer therapeutic targets in future.

## INTRODUCTION

Hepatoblastoma (HB) is the most common malignant liver tumor in children, accounting for approximately 50% of pediatric hepatic-related cancers [1]. The incidence of HB in children with age <15 is about one per million (1/1,000,000), and nearly 20% of those patients already have a synchronous metastasis at the first diagnosis. Despite recent advances in treatment, such as surgical resection, adjuvant chemotherapy, and liver transplantation, the prognosis in advanced HB stages still remains poor [2, 3]. It has been accepted that HB are composed of a variety of tumors deriving from different immature liver precursors, including hepatocytes, biliary, and other epithelial or mesenchymal cells, which caused significant tumor heterogeneity [4, 5]. For decades, researchers have observed the varying clinical outcomes in patients with different histological subgroups [5–9]. In addition, other parameters, such as tumor stage, distant metastasis, multifocality, patient age and birth weight, have also been reported to be associated with prognosis [10, 11]. However, the underlying mechanisms remains unknown. A thorough understanding of molecular mechanisms regarding tumor progression is essential for exploring effective therapeutic targets against HB.

Studies have revealed that only a small proportion (1%–2%) of the genome encodes proteins, and the majority of the mammalian genome encodes plenty of non-coding RNAs [12–14]. As an important member of the non-coding RNAs, microRNAs (miRNAs), referred to 18-25 nucleotides, have been clearly demonstrated to regulate a variety of cellular processes via recognizing the 3’-untranslated regions of specific mRNAs and suppressing the expression of target genes [15]. Long non-coding RNAs (lncRNAs), another sort of non-coding RNAs with more than 200 nucleotides, are found to play pivotal roles in chromatin remodeling, transcription regulation and post-transcriptional mRNA processing [16–19]. Recently, increasing studies reported that lncRNAs could also function as competing endogenous RNAs (ceRNAs) by competitively binding to miRNAs through their miRNA response elements (MRE) [20]. These ceRNAs usually share MRE with other coding transcripts and therefore act as sponges for that cluster of miRNAs, protecting the targeted mRNA transcripts from degradation [21]. Although the functional mechanism that lncRNAs act as ceRNAs leading to miRNA deregulation has been observed in multiple malignancies, the lncRNA-miRNA-mRNA network in HB is far from being fully investigated.

In the present study, we performed a comprehensive analysis based on mRNA, lncRNA and miRNA expression profiling data derived from 50 HB patients and 5 controls, and identified differentially expressed mRNA, lncRNA and miRNA in HB. Next, we employed weighted gene correlation network analysis (WGCNA) and coexpression analysis to select the modules which are associated with tumor malignant phenotypes. Based on above results, we finally conducted several potential lncRNA-miRNA-mRNA networks in HB.

## RESULTS

### Identification of the malignant phenotype-associated mRNAs and lncRNAs in HB

**Figure 1:**
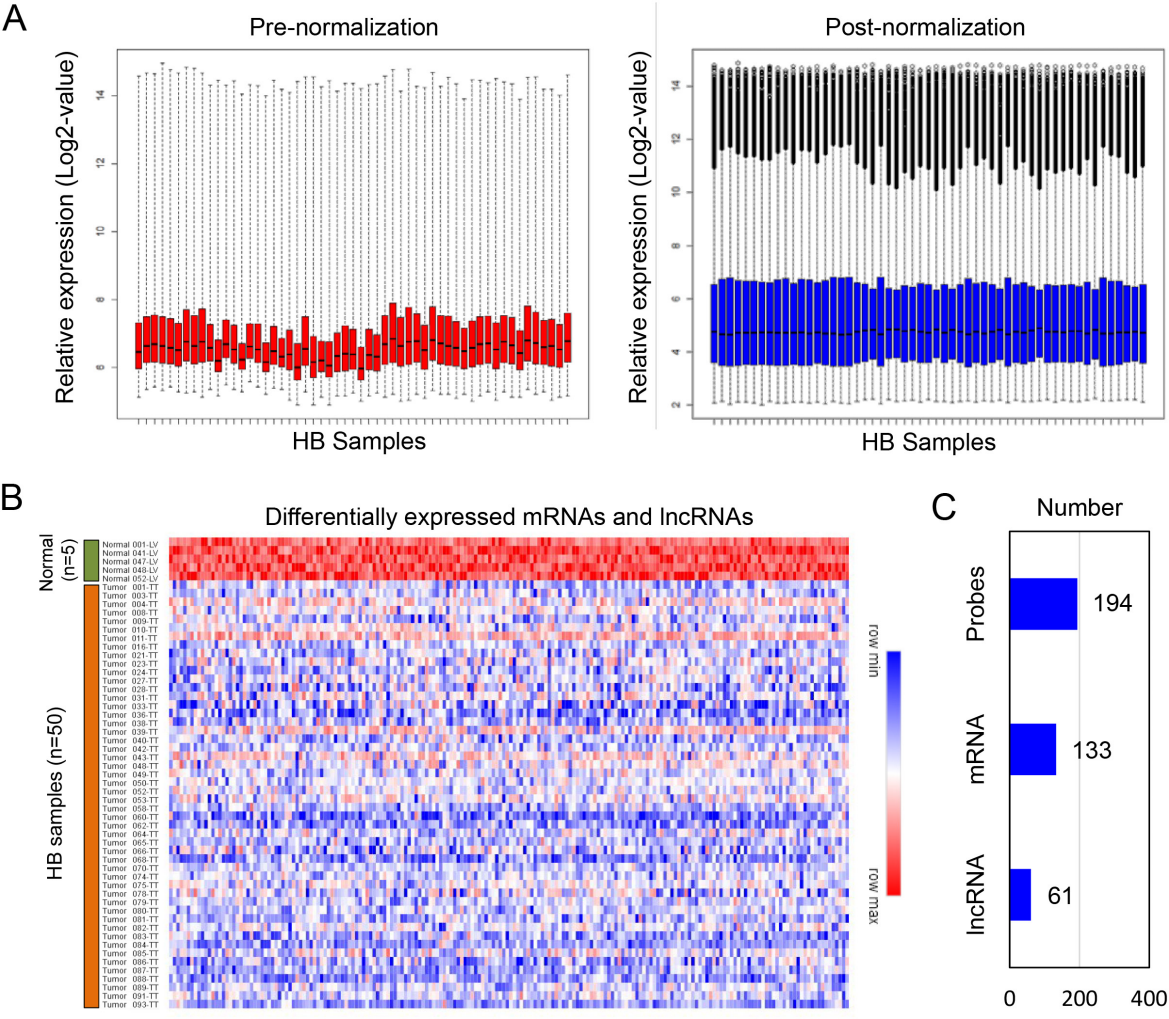
Identification of differentially expressed mRNA and lncRNAs from the HB dataset **(A)** Normalization of dataset GSE75271. **(B)** A total of 194 downregulated probes were picked up from HB patients. **(C)** Within the 194 downregulated probes, 133 mRNAs and 61 lncRNAs were identified.

GSE75271 was selected for identify differentially expressed mRNA and lncRNAs in HB patients [22]. As shown in Figure 1A, Then, we performed a probe level analysis of GSE75271 by linear models for microarray data (LIMMA). Those probes with *P*-value less than 0.05 and foldchange more than 2.0 were identified as differentially expressed probes. One hundred ninety-four probes were primary selected, which were all down-regulated in HB patients (Figure 1B). Next, 194 probes were annotated by Affymetrix microarray annotation files according to previous reported method [23]. Finally, we identified a total of 61 lncRNAs and 133 mRNAs from 194 differentially expressed probes (Figure 1C).

**Figure 2:**
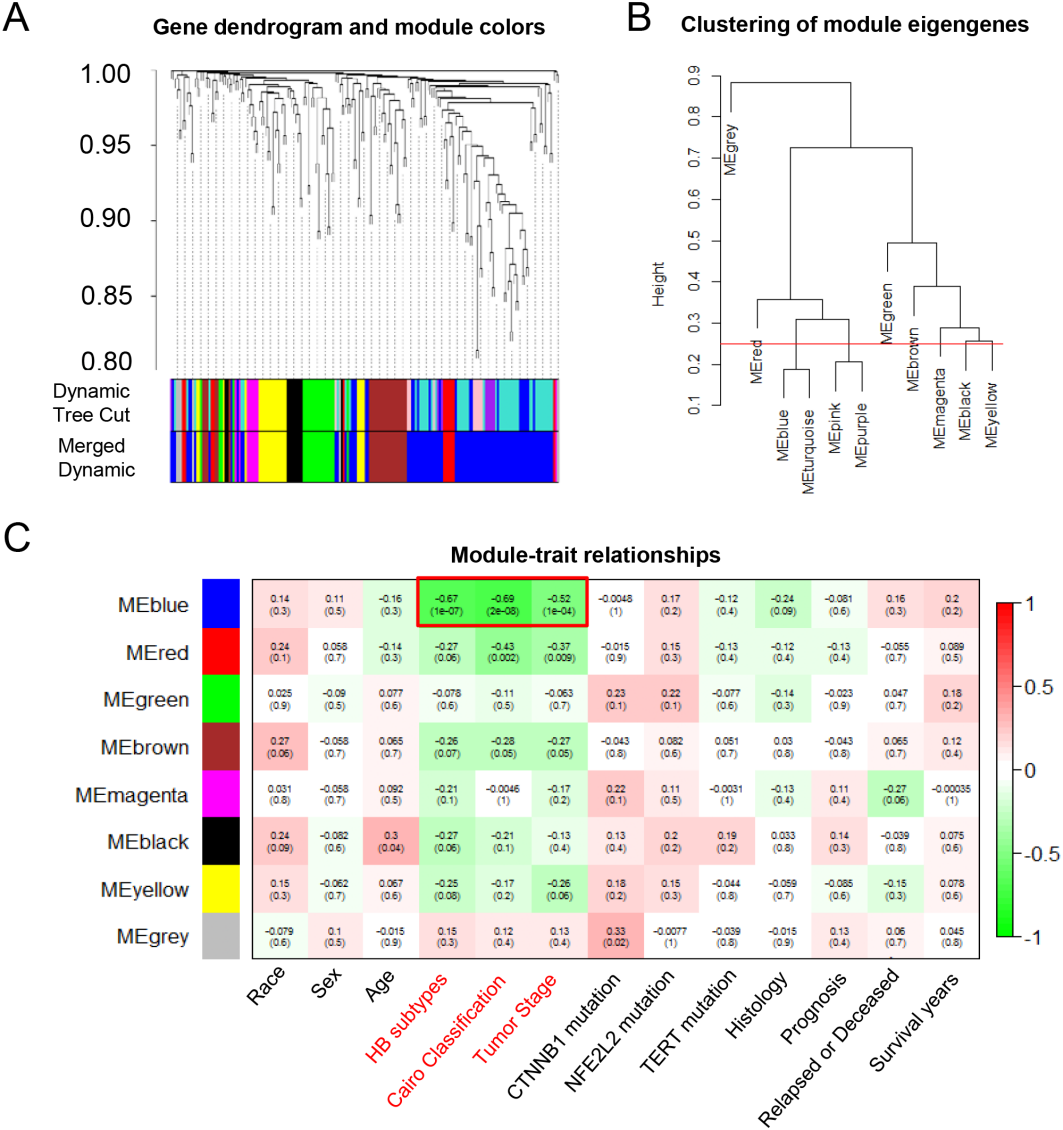
The WGCNA analysis of the malignant phenotype-associated mRNAs and lncRNAs **(A)** The cluster dendrogram of differentially expressed mRNAs and lncRNAs derived from the dataset GSE75271. **(B)** The cluster dendrogram of module eigengenes. **(C)** The module-trait relationship analysis between the 8 odules and clinical characteristics. ME: module eigengenes.

To further explore the malignant phenotype-associated mRNAs and lncRNAs, we performed a weighted gene correlation network analysis (WGCNA) (Figure 2A) and divided 194 differentially expressed probes into eight module eigengenes (ME), including MEgrey, MEred, MEblue, MEgreen, MEbrown, MEmagenta and MEblack (Figure 2B). Next, we employed module-trait relationship analysis to conduct the association between eight MEs and 13 clinical traits (including race, sex, age, HB subtypes, Cairo classification, tumor stage, *CTNNB1*, *NFE2L2* and *TERT* mutation status, histological types, prognosis, tumor recurrent status and survival time). Interestingly, we found that one module (MEblue) was negatively associated with HB subtypes, Cairo classification and tumor stage, with *P*-values of 1x10^-7^, 2x10^-8^ and 1x10^-4^, respectively (Figure 2C). The blue module contained a total of 66 mRNAs and 22 lncRNAs, which were selected as malignant phenotype-associated mRNAs and lncRNAs for the further analysis (Table 1).

**Table 1:** Identification of HB-associated mRNAs, miRNAs and lncRNAs

HB-associated mRNAs			HB-associated miRNAs	HB-associated lncRNAs
AC005523.3	GPRC5C	REPS2	NR_045387	hsa-miR-106b
ADHFE1	GSTA1	RET	NR_024548	hsa-miR-130a
AKR7A3	GSTZ1	RIPK4	NR_027005	hsa-miR-130b
ALDH6A1	HAO2	RNF144B	NR_040515	hsa-miR-17
AMBP	INSIG1	RPS6KA4	NR_102317	hsa-miR-18a
APBB1IP	KMO	SAR1B	NR_102357	hsa-miR-19a
APOA1	KNG1	SDC1	XR_171896	hsa-miR-19b
CBS	KRT81	SEBOX	XR_245338	hsa-miR-20a
CD53	LCAT	SLC22A7	XR_427361	hsa-miR-25
CDC37L1	LGR4	SLC7A2	XR_428373	hsa-miR-301a
CRAT	LPA	SMARCA4	XR_922928	hsa-miR-301b
CTAG2	MGAT5	STS	XR_923052	hsa-miR-449a
CTAGE15	MIR6778	SYDE1	XR_924107	hsa-miR-451
CYP1A2	MUT	TTC39C	XR_924945	
CYP2C18	NEUROG3	UGT1A1	XR_924990	
CYP4F2	NFKBIZ	VNN3	XR_929728	
DHTKD1	OAF	WFDC3	XR_931611	
EGFR	PCCB	ZFYVE28	XR_931899	
ETS2	PIGC	ZG16	XR_933428	
GAREM	PTCRA	ZNF584	XR_935074	
GLYAT	RARG	ZSCAN5A	XR_946696	
GPLD1	RDH16	ZYG11A	XR_949848	

### Identification of the malignant phenotype-associated miRNAs in HB

**Figure 3:**
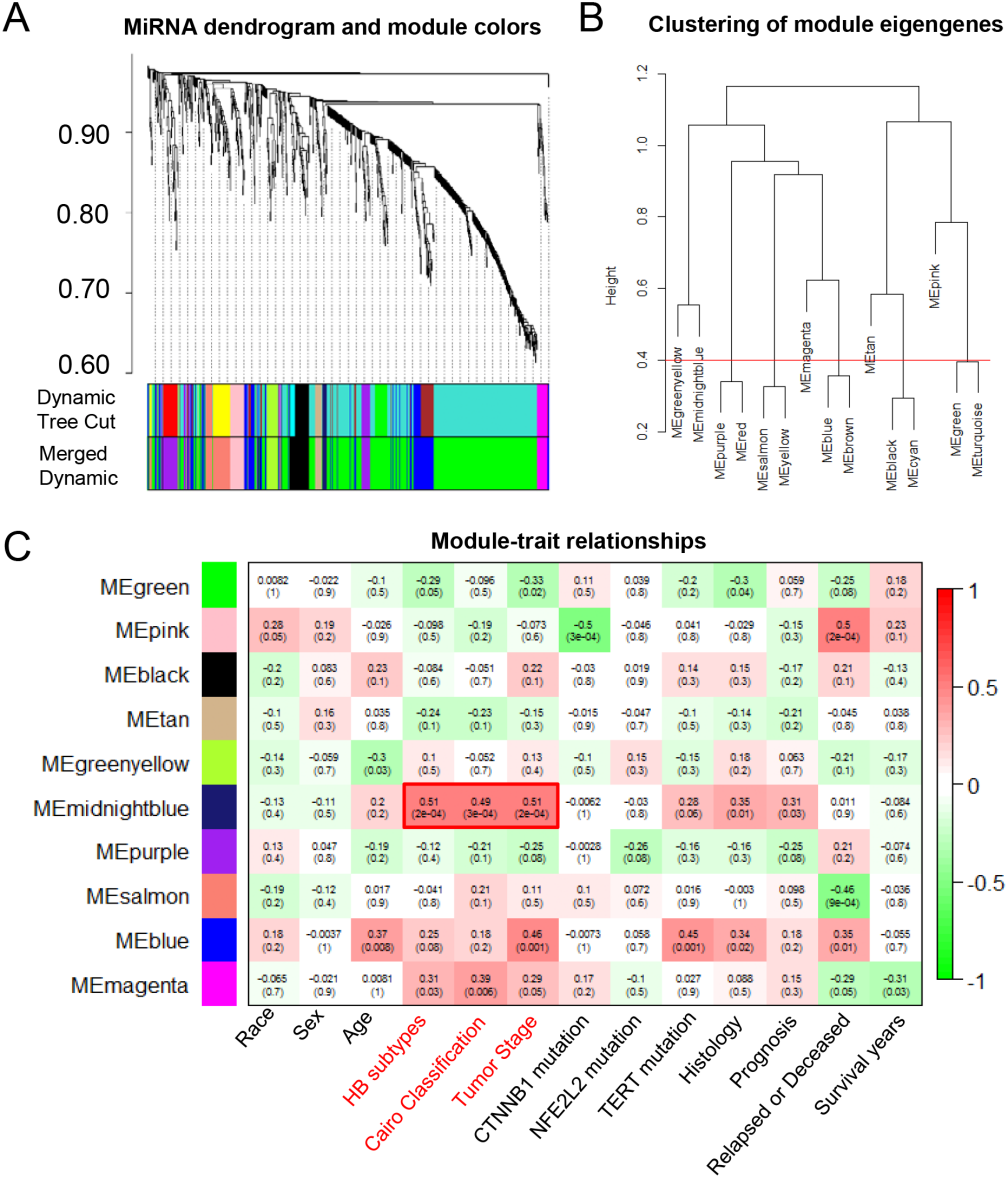
The WGCNA analysis of the malignant phenotype-associated miRNAs **(A)** The cluster dendrogram of differentially expressed miRNAs derived from the dataset GSE75283. **(B)** The cluster dendrogram of module eigengenes. **(C)** The module-trait relationship analysis between the 10 modules and clinical characteristics. ME: module eigengenes.

We also identified tumor-associated miRNAs using WGCNA method based on GSE75283, which was miRNA profiling dataset derived from the same HB patients as GSE75271 (Figure 3A). Similarly, we divided 887 miRNAs into ten MEs, including MEgreen, MEpink, MEblack, MEtan, MEgreenyellow, MEmidnoghtblue, MEpurple, MEsalmon, MEblue and MEmagenta (Figure 3B). Interestingly, the module-trait relationship analysis revealed that MEmidnoghtblue was positively associated with HB subtypes, Cairo classification and tumor stage, with *P*-values of 2x10^-4^, 3x10^-4^ and 2x10^-4^, respectively (Figure 3C). The midnoghtblue module contained 13 miRNAs, which were identified as malignant phenotype-associated miRNAs (Table 1).

### Coexpression analysis of malignant phenotype-associated molecules

**Figure 4:**
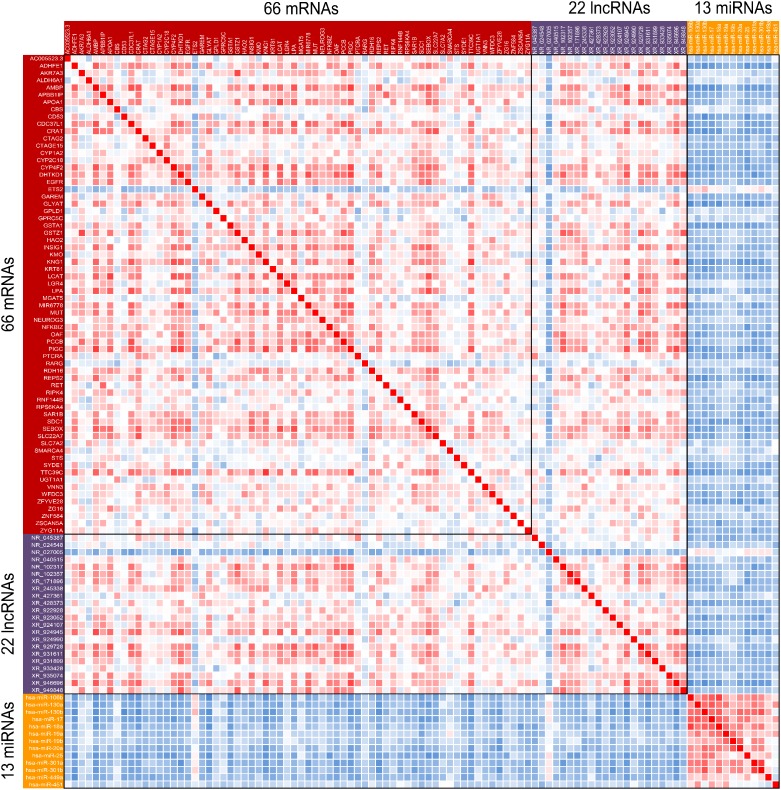
Coexpression analysis of malignant phenotype-associated molecules The heatmap was presented by correlation coefficient between each pair of malignant phenotype-associated molecules. Range of colors (red to blue) shows the correlation coefficient (high to low).

**Figure 5:**
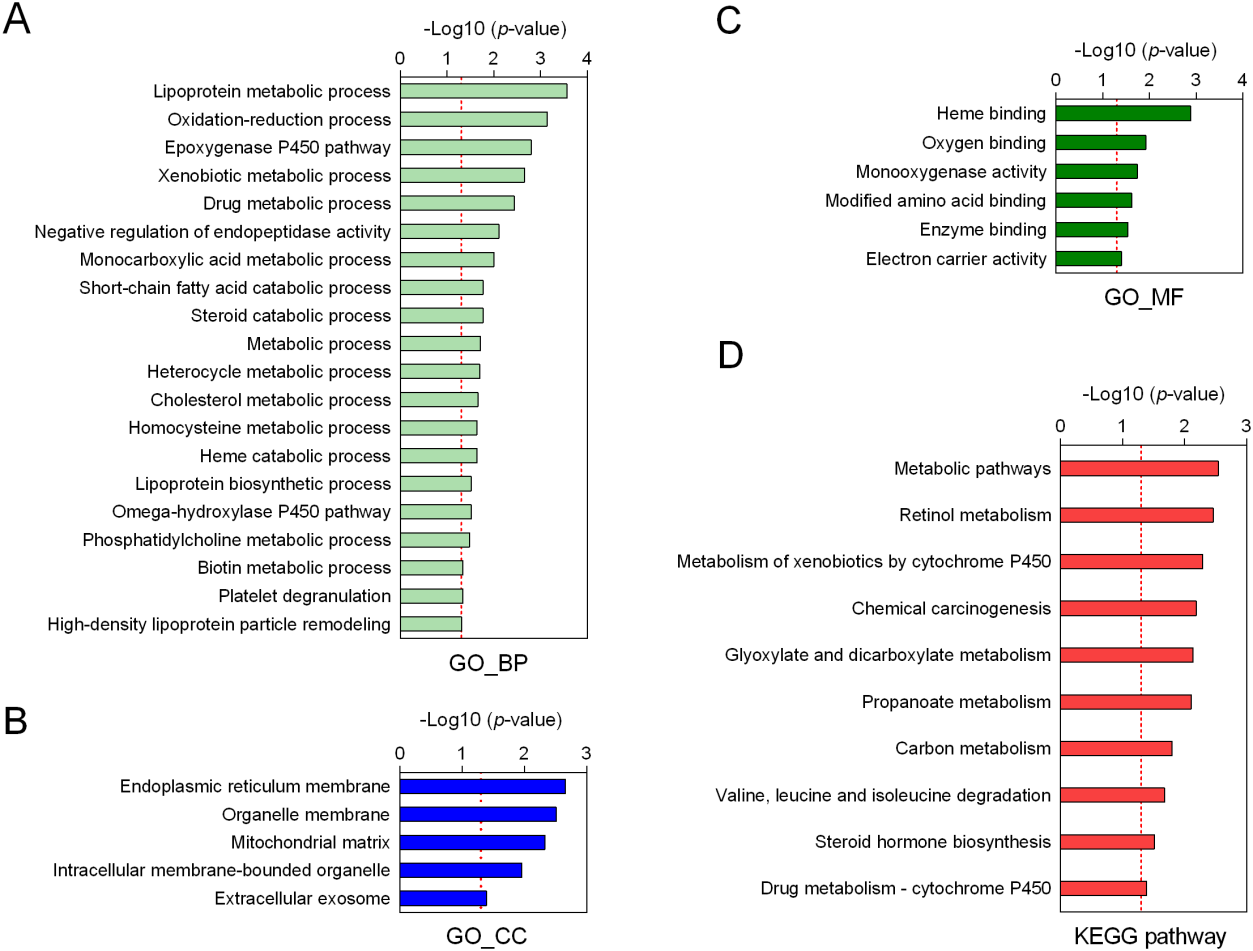
Function annotation of malignant phenotype-associated molecules Gene ontology analysis of biological processes **(A)**, cellular components **(B)** and molecular function **(C)** of those molecules. **(D)** The KEGG pathway enrichment analysis.

To explore the possible relationship between malignant phenotype-associated molecules, including mRNAs, lncRNAs and miRNAs, we performed coexpression analysis. As shown in Figure 4, we found that the lncRNAs were positively associated with nearly almost mRNAs. Interestingly, we also found that identified miRNAs were negatively associated with both mRNAs and lncRNAs. These results suggested that these mRNAs, lncRNAs and miRNAs might form lncRNA-miRNA-mRNA network, which affected the progression of HB patients. In order to predict biological function of above identified lncRNA-miRNA-mRNA network, we performed Gene ontology (GO) and Kyoto Encyclopedia of Genes and Genomes (KEGG) pathway enrichment analysis. Biological process analysis suggested these molecules were mainly involved in metabolic biological processes, such as lipoprotein metabolic process and oxidation-reduction process (Figure 5A). Cellular components analysis showed that they were located at endoplasmic reticulum (ER) membrane, the latter of which played a critical role in liver metabolism (Figure 5B). Besides, molecular function analysis also showed these molecules mostly belonged to heme-binding and oxygen-binding protein families (Figure 5C). KEGG pathway analysis further revealed that these malignant phenotype-associated molecules might be involved in regulating the metabolic related pathways to influence carcinogenesis and tumor progression (Figure 5D).

### Construction of lncRNA-miRNA-mRNA network in HB

**Figure 6:**
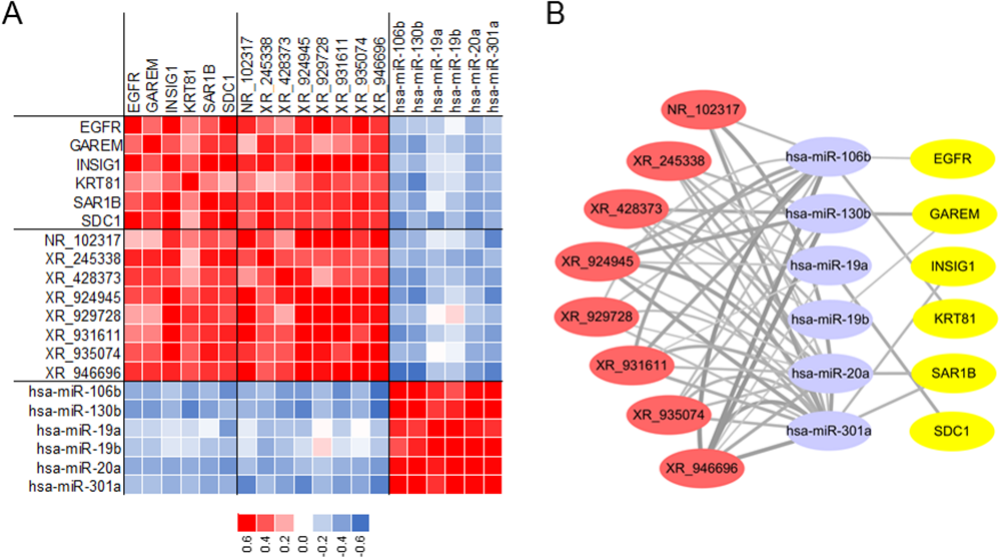
Construction of lncRNA-miRNA-mRNA network in HB **(A)** Selected 6 mRNAs, 8 lncRNAs and 6 miRNAs for network. **(B)** Predicted lncRNA-miRNA-mRNA network in HB.

We further conducted lncRNA-miRNA-mRNA network in HB based on the coexpression analysis of malignant phenotype-associated molecules. The construction of lncRNA-miRNA-mRNA network included three steps: (a) candidate malignant phenotype-associated lncRNAs, miRNAs and mRNAs were selected according to WGCNA results; (b) the correlation coefficients between lncRNAs, miRNAs and mRNAs were calculated based on their corresponding profiling data; (c) targets of miRNAs were predicted from Targetscan (http://www.targetscan.org/). Finally, a total of 6 miRNAs (hsa-miR-106b, hsa-miR-130b, hsa-miR-19a, hsa-miR-19b, hsa-miR-20a and hsa-miR-301a), 8 lncRNAs (NR_102317, XR_245338, XR_428373, XR_924945, XR_929728, XR_931611, XR_935074 and XR_946696), and 6 mRNAs (*EGFR, GAREM, INSIG1, KRT81, SAR1B* and *SDC1*) were selected to conduct lncRNA-miRNA-mRNA network. Heatmap was conducted to present the correlation coeffecient between molecules (Figure 6A), and Cytoscape were used for visualization of lncRNA-miRNA-mRNA network (Figure 6B).

## DISCUSSION

HB is a common malignant hepatic tumor in children and the prognosis varies among different categories. To date, there are limited effective methods to treat HB. Hence, it’s essential to explore underlying molecular mechanism of HB. In the present study, we for the first time identified the malignant phenotype-associated lncRNAs, mRNAs and miRNAs, which were associated with HB subtypes, Cairo Classification and tumor stage. Next, we conducted the lncRNA-miRNA-mRNA network based on their expression patterns to provide a molecular explanation for HB patients.

With the advancement of whole-genomic sequencing technologies, lncRNAs have attracted increasing attention [24]. Amount studies have reported that aberrant lncRNA expressions could serve as novel molecular biomarkers in the cancer diagnosis and prognosis prediction [25, 26]. LncRNAs could function as oncogenes, regulating alternation of key signaling pathways, and promoting tumor growth and metastasis [27, 28]. Recently, the miRNA sponge role of lncRNAs and lncRNA-miRNA-mRNA network have been widely accepted [20]. For instance, lncRNAs SPRY4-IT1 could sponge miR-101-3p and consequently increase EZH2 expression to promote cellular proliferation and metastasis of bladder cancer cells [29]. LncRNA-UCC was found to promote colorectal cancer progression by sponging miR-143 [30], and lncRNA-CCAT1 promotes hepatocellular carcinoma progression by sponging let-7 [31]. However, whether the lncRNA-miRNA-mRNA network plays an important role in HB patients have not been fully explored. A recent study reported that TUG1/miR-34a-5p/VEGFA network was involved in regulating hypervascularity and hepatoblastoma progression [32]. In the present study, we, for the first time, explored malignant phenotype-associated lncRNAs, miRNAs and mRNAs in HB by using WGCNA methods, respectively. A total of 22 lncRNAs, 13 miRNAs and 66 mRNAs were identified, which were strongly associated with HB subtypes, Cairo classification and tumor stage. These findings may provide us potential biomarkers and/or anti-cancer targets in future.

One hallmark of cancer is the metabolic reprogramming in cancer cells, including glycometabolism, lipid metabolism and amino acid metabolism [33–35]. In HB cell lines HepG2, Hep3B and HuH-6, the metabolic reprogramming has been defined as one important hallmark and contributes to tumor progression [36]. Several driver genes of HB, such as β-catenin, YAP and c-Myc [37, 38], could also affect intracellular energy metabolism. Moreover, it was also be found that that molecular targeting of mitochondrial metabolism holds promise as a novel and effective therapeutic approach for HB [39]. In the present study, we also performed pathway enrichment analysis of above identified molecules. Our results revealed that most molecules associated with HB classification and tumor stage belonged to metabolic pathway, indicating a close interaction between metabolic pathways and HB malignant phenotypes.

In this study, we also conducted the lncRNA-miRNA-mRNA network consisting of 6 miRNAs, 8 lncRNAs and 6 mRNAs, which was associated with HB classification and tumor stage. In this conducted network, we identified two genes in epidermal growth factor (EGF) signaling, *EGFR* and *SDC1* [40], acting as effector genes. Recently, a whole transcriptome analysis based on HB patients suggested that aberrant EGF signaling was associated with HB classification [41]. The loss of EGFR signaling members were shown to be more present in less differentiated embryonal and undifferentiated small cells subtypes of HB. Therefore, our findings might also provide evidence for explaining the underlying molecular variation of different HB subtypes.

In conclusion, we identified malignant phenotype-associated lncRNAs, miRNAs and mRNAs by employing WGCNA method. Mechanistically, these molecules might promote the malignant phenotypes of HB via regulating metabolic pathways. Moreover, we also conducted a lncRNAs-miRNAs-mRNAs network based on above findings. To our best knowledge, this is the first comprehensive study investigating lncRNA-miRNA-mRNA network in HB. If validated, our findings might provide evidence for exploring anti-cancer target in HB patients.

## MATERIALS AND METHODS

### Microarray data

Gene and miRNA profiling data of HB patients were searched from the Gene Expression Omnibus (GEO, http://www.ncbi.nlm.nih.gov/geo), which is a public database containing freely available profiling datasets. We finally selected two datasets for bioinformatic analysis, including GSE75271 (mRNA profiling data based on Affymetrix Human Genome U133 Plus 2.0 Array platform) and GSE75283 (miRNA profiling data based on Agilent-029297 Human miRNA Microarray v15 platform), which were derived from the same 50 HB patients and 5 controls [22]. All raw data were downloaded from GEO database.

### Data normalization and probe annotation

The raw data of GSE75271 was stored as probe-level CEL files, and was quantile normalized using Robust Multi-array Average (RMA) method. After data normalization, expression level of each probe in GSE75271 was obtained. The probe sequences with corresponding Affymetrix probe IDs were downloaded from the Affymetrix website (http://www.affymetrix.com). The annotation of lncRNA transcripts were then performed according to previous reported method [23]. A total of 8240 lncRNA transcripts were generated with RefSeq transcript IDs. The probe ID-centric gene expression profile was also generated according to Affymetrix annotation files.

### Weighted gene correlation network analysis

WGCNA is an algorithm for constructing a co-expression network, defined by the similarity of gene co-expression [42]. In data processing, the genome-wide gene expression data was initially filtrated with measuring the consistency of gene expression profiles by Pearson correlation, then we utilized the power adjacent function to Pearson correlation matrix to transform data into weighted gene co-expression networks. Network module represents a cluster of closely interconnected genes. Finally, the adjacency matrix, a measurement of topology similarity, is converted into the topological overlap matrix (TOM), and modules are detected by cluster analysis [43].

### Pathway enrichment analysis

GO analysis is freely available for users in the annotation and biological properties of genes, gene products and sequences [44]. KEGG is a knowledge base for systematic analysis of gene functions in terms of the networks of genes and molecules [45]. We used GO analysis and KEGG analysis to identify the function of the aberrantly expressed candidate lncRNAs-associated genes. The *P*-value of each enriched pathway was assigned with -log10 transformation. *P*<0.05 (-log10(*P*-value)=1.30) was considered as statistically significant.

### Statistical analysis

All data were analyzed by R software 3.4.1 (https://www.r-project.org/). For the pair of molecule *i* and *j*, Pearson correlation coefficient was computed. *P* < 0.05 was considered statistically significant.

## Abbreviations

ceRNAs: competing endogenous RNAs
ER: endoplasmic reticulum
EGF: epidermal growth factor
GEO: Gene Expression Omnibus
GO: Gene ontology
HB: Hepatoblastoma
KEGG: Kyoto Encyclopedia of Genes and Genomes
LIMMA: linear models for microarray data
lncRNA: long non-coding RNAs
miRNA: microRNAs
MRE: miRNA response elements
ME: module eigengenes
RMA: Robust Multi-array Average
TOM: topological overlap matrix
WGCNA: weighted gene correlation network analysis
